# A Pragmatic Randomized Controlled Trial to Increase PrEP Uptake for HIV Prevention: 55-Week Results From PrEPChicago

**DOI:** 10.1097/QAI.0000000000002518

**Published:** 2021-10-23

**Authors:** John A. Schneider, Lindsay Young, Arthi Ramachandran, Stuart Michaels, Hildie Cohen, Ishida Robinson, Leigh Alon, Brandon Hill, Sarah Nakasone, Mario Balenciaga, Darnell Motley, Alida Bouris, Aditya Khanna, Matthew Ferreira, Thomas Valente, Phillip Schumm

**Affiliations:** a University of Chicago, Chicago, IL;; b NORC at the University of Chicago, Chicago, IL; and; c University of Southern California, Los Angeles, CA.

**Keywords:** PrEP, social network analysis, Black MSM

## Abstract

**Objectives:**

We tested preliminary efficacy of a peer change agent type I network intervention to increase pre-exposure prophylaxis (PrEP) linkage to care among network members connected to young Black men who have sex with men.

**Design:**

Parent study is a pragmatic randomized controlled trial with 110 weeks of total follow-up. Interim midpoint analyses are performed here using participant data before crossover assignment at 55 weeks.

**Methods:**

We randomly assigned 423 participants in Chicago to receive the network intervention, an opinion leader workshop with telephonic booster sessions, versus a time-matched control from 2016 to 2018. The consolidated surrogate outcome was PrEP referral and linkage to clinical care among network members connected to study participants and was collected from independent administrative data.

**Results:**

Each study participant in the trial (n = 423) had on average 1822 network contacts who could be eligible for PrEP referral and linkage. During the 55-week observation period, PrEP referral was most likely to occur within 3 days of an intervention session compared to control [odds ratio (OR) 0.07 (0.02–0.013); *P* = 0.007] resulting in 1–2 referrals of network members per session. Network members with referral or linkage were more likely to be connected to study participants in the intervention arm than the control condition [aOR 1.50 (1.09–2.06); *P* = 0.012].

**Conclusions:**

A peer change agent type I network intervention is preliminarily effective at diffusing PrEP through a network of individuals highly susceptible to HIV over 55 weeks. This low-intensity intervention demonstrated network-level impact among populations that have experienced limited PrEP care engagement in the United States.

## Abstract 2

Supplemental Digital Content is Available in the Text.

HIV prevention in the form of pre-exposure prophylaxis (PrEP) has demonstrated efficacy with FDA approval in 2012 for use in adults as once daily tenofovir/emtricitabine.^1^ More recently, there have been several PrEP advances, particularly in the timing of dosing,^2^ extension to individuals aged 13–17 years,^3^ and a newly efficacious product.^4^ During this timeframe, studies have documented limited PrEP awareness, referral, and linkage to care among several populations experiencing high HIV incidence, most notably young Black men who have sex with men (YBMSM).^5–8^ Broad implementation strategies are therefore required to improve the uptake of PrEP within most impacted populations.^9,10^

Decades of social diffusion research underscores the impact of social relationships in the adoption and spread of novel health interventions in a population.^11^ Diffusion of biomedical innovation has been described in landmark observational studies, such as the context of family-planning methods^12,13^ and in the diffusion of prescribing practices through provider networks.^14^ Underlying all diffusion processes is that innovation will spread to others through interpersonal networks.^11^ More recently, advances in network interventions have been proposed to accelerate this diffusion process from a public health framework. Network interventions, whether they aim to diffuse biomedical or behavioral innovations, are categorized as 4 types.^15^ The most common approach is a type 1 peer change agent approach where individuals are selected based upon their popularity or socially advantageous network position and trained to deliver innovation to others within their networks. This approach has been used previously in many other contexts including diabetes self-management, treatment of myocardial infarction, and vaginal delivery after previous cesarean section.^16–19^

Here, we describe the midpoint 55-week impact of PrEP Chicago, a type 1 network intervention. PrEP Chicago was developed to engage YBMSM—a population estimated to have a 1 in 2 chance of acquiring HIV in their lifetime.^9^ If successful, the intervention would be an important implementation strategy for ending the epidemic in the United States.^20^

## METHODS

### Study Population

The PrEP Chicago intervention recruited YBMSM living in Chicago and adjacent neighborhoods from 2016 to 2018. Cook County (Chicago) is a joint NIH/DHSS designated ending the epidemic jurisdiction and ranks third in total HIV burden in the United States.^21^ During the study period, the citywide PrEP4Love PrEP information campaign was in existence and included a warmline that provides information on PrEP and referral to PrEP care throughout the city.^22^ Accessible PrEP care was available throughout Chicago, including a network of sexual health clinics that provide the majority of PrEP care in the Midwest irrespective of insurance status.^7^

Participants were eligible if they were 18–35 years of age, identified as Black/African American, were male sex at birth, had sex with a man in the past 12 months, and, because the intervention emphasizes social media as a communication tool, had an active Facebook profile. Once deemed eligible, individuals were assigned randomly to 1 of the 2 treatment conditions. Those randomized to the intervention received the PrEPChicago intervention over 55 weeks and participants randomized to the time-matched control condition received an intervention over the same period. Randomization was computer generated in 1:1 intervention to control ratio and allocated immediately by study staff after participant consent.

### Study Procedures

The findings presented here represent analysis of the PrEPChicago intervention, a 2-arm pragmatic randomized trial (ClinicalTrials.gov number, NCT 02896699).^23^ The findings presented here include analysis before the 55-week intervention switch (baseline intervention receives control and baseline control receives intervention). PrEPChicago was approved by the institutional review board at the University of Chicago. Recruitment of study participants occurred between March 2016 and March 2017 using respondent-driven sampling,^24^ a variant of snowball sampling that draws on referrals, beginning with a set of initial “seeds” that meet study eligibility. Enrolled seeds are known members of the community who are asked to recruit members of their social networks into the study. After enrollment, these new participants were invited to recruit peers, and the process continued until the recruitment target was reached.

Data were collected from all participants at baseline and 12-month follow-up. To measure the PrEP diffusion outcome, digital network data were collected from each participant using Facebook's data download feature. Lists of a participant's Facebook friends at baseline were retained for analysis to match with independently collected administrative data of community members referred for PrEP or making a PrEP appointment. Data protections to secure third-party (nonparticipant) identities were established, and a waiver of consent from the IRB for third-party (nonparticipant) network members was obtained, given the minimal risk to these individuals.

### Intervention Condition

The PrEP Chicago intervention aims to develop a participant's knowledge about PrEP and build skills around PrEP communication and motivation to engage peers in the PrEP care continuum.^23^ The intervention is composed of 2 parts: (1) a half-day, small group training workshop led by intervention staff and (2) a series of check-in calls (or “boosters”) between intervention staff and participants. The intervention workshop adapts the peer educational and mentoring program developed as part of the HIV Prevention Trials Network^25,26^ and is divided into 4 modules: (1) HIV facts and myths; (2) background on PrEP; (3) role playing conversations about motivating peers to engage in PrEP care; and (4) leveraging social media to spread awareness about PrEP. The third and fourth modules develop participants' PrEP engagement communication skills to increase their effectiveness as peer change agents. Participants are trained to motivate their peers to make a PrEP appointment through a number of PrEP clinics and the citywide PrEPline that refers clients to PrEP care.^22^ After the baseline workshop, trained staff administered a total of 8 monthly booster calls with each participant, each lasting 10–20 minutes. Boosters were designed to help participant peer change agents to devise personalized conversational strategies for approaching peers and to troubleshoot communication barriers.

### Time-Matched Control Condition

Participants were randomized to the intervention condition just described or to a minimal contact attention control condition.^27^ The attention control condition consisted of a sexual risk assessment workshop, whereby participants wrote and discussed fictional narratives about what they believe constituted low, medium, and high HIV/STI risk scenarios.

### Consolidated Surrogate Outcome

Outcome measurement in such a pragmatic randomized controlled trial^28^ is complicated by the fact that it is impossible to determine with certainty which participant(s) may have influenced a specific individual to engage in PrEP care activities, such as calling the PrEPline or making a clinic appointment. Moreover, information obtained by asking either party directly would likely have been incomplete and subject to reporting biases. In fact, over 80% of the data field “who recommended you to the PrEPline” was found to be missing in the citywide PrEPline database, which limited the original planned analysis. Thus, for this interim analysis, a new consolidated surrogate outcome was developed for *network members* not enrolled in the study who were linked to study participants over the observation period: (1) active referral to PrEP care; or (2) attendance of a first PrEP care appointment. The first source of data used to infer PrEP care engagement of network members was the citywide PrEPline database restricted to all referrals made to PrEP care over the 55-week study period, which were initiated by community members. The second source of information was electronic medical record data of all first PrEP appointments made by patients at the largest PrEP serving clinic system in Chicago over the 55-week study period. The use of warmline and clinic appointment measures collected independently of each other and independent of the study provide greater objectivity than other self-report measures typically used in diffusion of innovation studies.

To link the referral and appointment databases to the network members of study participants, a pool of network members not enrolled in the study who are linked to study participants was required. Facebook friend lists were abstracted from each study participant using the manual data download feature offered by Facebook, which allows a Facebook user to download all or parts of their data, including Facebook friends. Independent linkage of Facebook friends to the PrEPline and clinic data was conducted to determine which specific network members from this pool were either referred to PrEP care or attended a PrEP appointment, after enrollment. An honest broker not affiliated with the study and blinded to treatment condition matched Facebook aliases of study participant friends with the citywide PrEPline and clinic data. Referrals and appointments that were connected to both intervention and control participants were counted toward a specific arm based upon having more ties to a given arm; when equal ties across both arms were observed, such referrals/appointments were excluded from analyses.

### Statistical Analyses

Two separate analyses were performed: (1) a timing analysis of PrEP referrals after intervention sessions; and (2) a primary comparison of intervention and control conditions. For the first approach, a timing analysis was performed to determine the relationship between intervention and the PrEP referral outcome. If the intervention was not effective in increasing the number of successful referrals, then there should be no association between the dates on which the intervention was delivered and the dates on which referrals were made. To examine this relationship, we modeled the number of PrEPline referrals initiated per day by the citywide PrEPline as a function of the number of workshops and boosters delivered on that day and the days immediately preceding, adjusting for weekly differences in the overall intensity of calls and in the number of workshops/boosters over the course of the study that might confound the causal association between them. We fit a mixed-effects negative binomial model^29^ to the data collected from the 55-week enrollment period. The model was fit in Stata 15.1^30^ using maximum likelihood with mean-variance adaptive Gauss–Hermite quadrature. Estimates are presented, together with approximate 95% confidence intervals obtained by inverting the corresponding Wald test.

For the second analysis, if the intervention was not effective, then the likelihood of Facebook friends referred for PrEP or attending a PrEP appointment should be unrelated to whether the participant was assigned to the intervention or the control condition. To test this hypothesis, a conditional logistic regression^31^ was fit to the likelihood of Facebook friendship ties existing between study participants and those with PrEP referral or PrEP appointment as a function of participants' group assignments, adjusting for each participant's total number of Facebook friends and whether the person was referred into the study by another participant. Specifically, each PrEP referral or clinic visit among the pool of Facebook friends was treated as an independent observation for which the set of possible ties consisted of all study participants who had been recruited at the time of the referral (or visit). The model including all possible ties was fit using only those potential ties involving participants who had been recruited to the study within the first 12 weeks after intervention or control, to account for the possibility that the effect of the intervention was greatest within the first few weeks after the initial in-person session. Estimated odds ratios are presented, together with corresponding 95% confidence intervals.

## RESULTS

### Study Subjects

**FIGURE 1.**
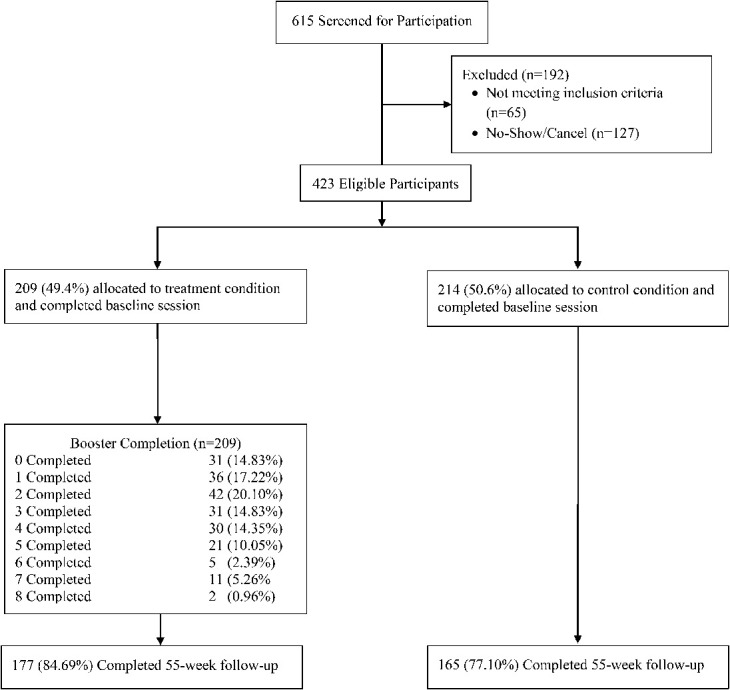
Enrollment and follow-up of study participants before 55-week intervention sequence switch.

From March 2016 to March 2017, 423 individuals were recruited and randomized, with 209 assigned to the intervention group and 214 to the control group (Fig. 1). Demographic and other characteristics of the study participants were equivalent by arm except for gender identity (Table 1). There were 365,346 unique Facebook network members connected to study participants from which the surrogate outcome was measured.

**TABLE 1.** Baseline Sociodemographic and Behavioral Characteristics Among PrEP Chicago Participants, Chicago, (2016–2018)

Characteristics	Intervention (%)* (n = 209)	Control (%) (n = 214)	*P*
Age, mean (SD)	26.1 (4.2)	25.7 (4.3)	0.28
Education			0.17
High school or less	141 (67.5)	165 (77.1)	
Post HS vocational certification	17 (8.1)	11 (5.1)	
Associate's/Bachelor's/Grad. degree	43 (20.6)	29 (13.6)	
Employment			0.31
Employed	109 (52.2)	92 (43.0)	
Not employed	81 (38.8)	99 (46.3)	
Disabled	6 (2.9)	7 (3.3)	
Gender identity			0.043
Male	193 (94.2)	184 (88.0)	
Female/transfeminine	7 (3.4)	20 (9.6)	
Other	5 (2.4)	5 (2.4)	
Sexual orientation			
Gay	135 (64.6)	123 (57.5)	0.55
Bisexual	46 (22.0)	62 (29.0)	
Straight	5 (2.4)	8 (3.7)	
Other	11 (5.3)	10 (4.7)	
HIV-Positive	92 (48.4)	87 (46.5)	0.71
Ever heard of PrEP			0.20
No	50 (23.9)	66 (30.8)	
Yes	156 (74.7)	143 (66.8)	
Experience taking PrEP			
No	180 (86.1)	186 (86.9)	
Yes	20 (9.6)	20 (9.4)	0.95
Facebook friends, mean (SD)	1810 (1394.3)	1859 (1503.4)	0.73

### Temporal Relationship of Intervention and PrEP Referral

**FIGURE 2.**
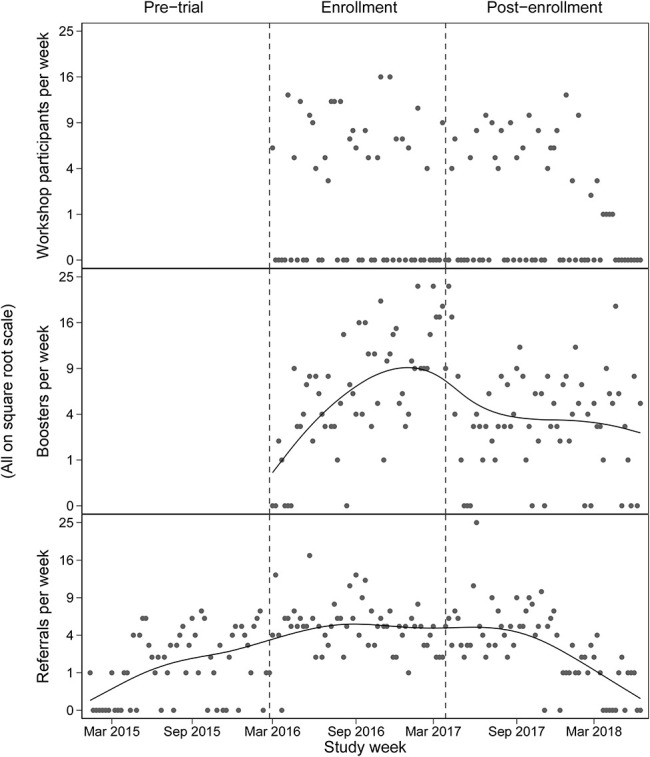
Number of participants completing an intervention workshop per week (Top); number of men completing a booster per week, with penalized spline smoother (Middle); weekly number of PrEPline referrals, with penalized spline smoother (Bottom).

Table 2 shows estimates from a mixed-effects negative binomial model fit to the daily number of citywide PrEPline referrals. The number of participants completing an intervention workshop was positively associated with the number of PrEPline referrals, both on the same day and for the next 2 days (by the third day, the effect was no longer evident). Thus, a workshop involving 5 participants translates into approximately one additional PrEPline referral over 3 days, assuming a background referral rate of 5.4 calls per week. By contrast, booster sessions were not associated with an increase in the number of referrals—either on the same day or for the next 3 days (Fig. 2). The purpose of Figure 2 is to demonstrate in the most transparent way possible how the provision of the study intervention (ie, workshops and boosters) was associated with changes over time in the mean number of PreP referrals/care visits.

**TABLE 2.** Daily Number of PrEPline Calls on the Number of Intervention Workshops and Boosters, Adjusting for Day of Week and Weekly Variation Over Midpoint 55 Weeks of Follow-up*

	Estimate (95% CI)	*P*
Daily workshops		
Same day	0.08 (0.02 to 0.13)	0.009
1 day lag	0.10 (0.04 to 0.15)	0.001
2 day lag	0.06 (0.00 to 0.12)	0.044
3 day lag	0.01 (−0.05 to 0.08)	0.663
Daily boosters		
Same day	−0.07 (−0.14 to 0.00)	0.054
1 day lag	0.05 (−0.03 to 0.13)	0.251
2 day lag	−0.01 (−0.07 to 0.05)	0.719
3 day lag	0.00 (−0.10 to 0.11)	0.953
Day of week (vs. Monday–Friday)		
Saturday	−1.80 (−2.55 to −1.05)	<0.001
Sunday	−2.50 (−3.53 to −1.46)	<0.001
Constant	−0.14 (−0.38 to 0.10)	0.249
Log (α)	−1.43 (−2.34 to −0.52)	
σ†	0.24 (0.09 to 0.64)	

* Negative binomial mixed-effects regression models.

† SD of the week-specific random effects.

### Intervention Effect on PrEP Referrals and Appointments

Over 55 weeks, 15 network members referred from the PrEPline and 50 with a PrEP referral or care visit were identified as having a network tie to at least one study participant who had been recruited before the PrEP referral/visit (ie, they were “Facebook friends”). Two-hundred and sixteen participants (119 intervention and 97 control) were tied to at least one PrEP engaged individual; these participants had a mean of 2.6 ties, a median of 2, and a range of 1–10. Video 1, Supplemental Digital Content, http://links.lww.com/QAI/B565 is a dynamic network visualization that shows the diffusion of PrEP through this network. Estimates from a conditional logistic regression model fit to the likelihood of network ties between PrEP referrals/clinic visits and each of the study participants recruited before the referral/visit are presented in Table 3. Over a 12-month period, the odds of a tie existing between a referral/PrEP visit and a participant in the intervention group were 50% higher [95% confidence interval (CI): 9% to 106%] than for participants in the control group.

**TABLE 3.** Likelihood of a Facebook Tie Between PrEPline Referral or First PrEP Clinic Appointment (n = 65) and Study Participant Over 55 Weeks of Follow-Up

	Covariate	OR*	95% CI	*P*
All ties over 12 months	Intervention vs. control	1.19	0.99 to 1.43	0.067
	Seed vs. recruit	1.46	1.19 to 1.79	<0.001
	No. of FB friends (thousands)	1.67	1.57 to 1.78	<0.001
Ties within 3 months of intervention	Intervention vs. control	1.50	1.09 to 2.06	0.012
	Seed vs. recruit	0.71	0.46 to 1.11	0.133
	No. of FB friends (thousands)	1.53	1.38 to 1.70	<0.001

Conditional logistic regression model.

## DISCUSSION

Results from this pragmatic randomized controlled trial at 55 weeks of follow-up demonstrate that the intervention was successful in generating PrEP referrals and linkage to first PrEP appointments. The results are robust with the use of 2 independently collected outcome measures as well as 2 separate analytic approaches to determine intervention effect. The 2 analytic approaches each yield evidence of intervention effectiveness over 55 weeks, an adequate time to examine PrEP linkage to care. When compared to control condition, the intervention yielded greater PrEP referral or first clinic appointment, an effect that was strongest within the first 3 months of the intervention. The analysis of referral timing indicates that unlike the workshops, the boosters did not increase the number of referrals over the next few days, although this does not address the possible value of the boosters in maintaining engagement over a longer period.

The estimated number of PrEPline referrals generated during the days immediately after the workshops likely underestimates the overall effect of the intervention—possibly by a large amount. This is due to the small observation window of less than a week that was required to keep potential effects separate from subsequent week interventions. By adjusting for week-to-week variation in PrEPline referral intensity and the number of workshops/boosters, the referral timing analysis focuses solely on the immediate effects of the intervention components, in an attempt to avoid spurious association due to factors throughout the study period that might have affected both the frequency of referrals or first PrEP appointments and the delivery of the intervention. However, the frequency of PrEPline referrals was higher during the study period than it had been before, and began to decline again once the workshops were nearing completion. Thus, it is likely that participants who received the intervention continued to influence network members throughout the study period. Such episodic influence would be nearly impossible, to measure without a mechanism for tracing the interactions between individual participants and PrEPline clients.

The use of Facebook data to measure social influence within the context of a PrEP intervention is important for pragmatic trials that measure downstream outcomes among individuals not enrolled in a study. However, apart from providing a convenient way to track the effect of the intervention, the Facebook results are also informative about potential mechanisms by which such influence occur. Specifically, they suggest the potential importance of Facebook as a means of communication within this community—one that may have real consequences for health-related behavior, which has also been demonstrated in other Facebook communication interventions.^32^ Furthermore, Facebook's move into dating services underscores the importance of interventions that include such traditional social media applications. Of course, as with the referral timing analysis, the Facebook results may capture only part of the intervention's overall effect because some referred network members or those making an appointment were not locatable on Facebook, appointments may be made at other clinic contexts, and referrals may be made in social contexts not reflected on Facebook. Despite this, diffusion of PrEP through networks could only be accelerated through both online and offline information and motivation.

There were a few limitations to the study. First, we were unable to observe direct communication and referral of network members for PrEP; however, an alternative explanation for the observed PrEP engagement is not available. Second, we did not measure onward diffusion of PrEP beyond the first-degree network members. Although this is possible and would potentially increase the impact of the PrEPChicago intervention more widely, we do know that secondary and tertiary network diffusion effects are much more limited and likely do not move much beyond the third degree. Finally, it is unclear as to whether why we did not observe an effect from the booster sessions. In addition, to several boosters being inadvertently skipped by study staff, it may be that the impact was lessened as the boosters were uncompensated sessions, which could have potentially contributed to limited participant enthusiasm and overall impact.

Diffusion of behaviors have also been observed for smoking cessation,^33^ adolescent substance use,^34^ and other HIV-prevention activities.^35–37^ The ability to reach larger portions of targeted populations^38,39^ while also overcoming socially derived barriers such as stigma and discrimination through peer leadership and support makes social network interventions powerful strategies for engaging communities most impacted by HIV in biomedical innovations like PrEP. PrEPChicago is thus one of several low-cost strategies that could be used to address HIV spread locally and regionally, such as in national DHHS ending the epidemic strategies. Given that the most impactful component from the interim analysis presented herein was the in-person group intervention, a single session is thus useful in diffusing innovation and represents a low-cost intervention that is potentially scalable. Final analysis will examine the durability of the intervention over 110 weeks for PrEP linkage as well as explore impact beyond linkage such as important PrEP persistence metrics. Future research should continue to explore how such interventions can be used to help end the domestic HIV epidemic, especially in settings where there is low PrEP awareness and access.
